# Characterisation of neonatal *Staphylococcus capitis* NRCS-A isolates compared with non NRCS-A *Staphylococcus capitis* from neonates and adults

**DOI:** 10.1099/mgen.0.001106

**Published:** 2023-10-04

**Authors:** Heather Felgate, Dheeraj Sethi, Kirsten Faust, Cemsid Kiy, Christoph Härtel, Jan Rupp, Rebecca Clifford, Rachael Dean, Catherine Tremlett, John Wain, Gemma Langridge, Paul Clarke, Andrew J. Page, Mark A. Webber

**Affiliations:** ^1^​ Quadram Institute Bioscience, Norwich Research Park, NR4 7UQ, Norwich, UK; ^2^​ Norwich Medical School, University of East Anglia (UEA), Norwich, UK; ^3^​ Norfolk and Norwich University Hospital (NNUH), NR4 7UY, Norwich, UK; ^4^​ Department of Pediatrics, University of Lübeck, Lübeck, Germany; ^5^​ Department of Pediatrics, University of Würzburg, Würzburg, Germany; ^6^​ Department of Infectious Diseases and Microbiology, University of Lübeck, Lübeck, Germany

**Keywords:** Antimicrobial Resistance, CRISPR, infection control, Late Onset Infection, microbiota, neonates, skin

## Abstract

*
Staphylococcus capitis* is a frequent cause of late-onset sepsis in neonates admitted to Neonatal Intensive Care Units (NICU). One clone of *S. capitis *, NRCS-A has been isolated from NICUs globally although the reasons for the global success of this clone are not well understood.

We analysed a collection of *S. capitis* colonising babies admitted to two NICUs, one in the UK and one in Germany as well as corresponding pathological clinical isolates. Genome analysis identified a population structure of three groups; non-NRCS-A isolates, NRCS-A isolates, and a group of ‘proto NRCS-A’ – isolates closely related to NRCS-A but not associated with neonatal infection. All bloodstream isolates belonged to the NRCS-A group and were indistinguishable from strains carried on the skin or in the gut. NRCS-A isolates showed increased tolerance to chlorhexidine and antibiotics relative to the other *S. capitis* as well as enhanced ability to grow at higher pH values. Analysis of the pangenome of 138 isolates identified characteristic *nsr* and *tarJ* genes in both the NRCS-A and proto groups. A CRISPR-cas system was only seen in NRCS-A isolates which also showed enrichment of genes for metal acquisition and transport.

We found evidence for transmission of *S. capitis* NRCS-A within NICU, with related isolates shared between babies and multiple acquisitions by some babies. Our data show NRCS-A strains commonly colonise uninfected babies in NICU representing a potential reservoir for potential infection. This work provides more evidence that adaptation to survive in the gut and on skin facilitates spread of NRCS-A, and that metal acquisition and tolerance may be important to the biology of NRCS-A. Understanding how NRCS-A survives in NICUs can help develop infection control procedures against this clone.

## Data Summary

All sequence data was submitted the NCBI SRA archive under the project number PRJNA751027, accession number SAMN30525685 - SAMN30525814.

**Impact Statement**

Neonates are highly vulnerable to infection and one clone of *S. capitis *, NRCS-A has been shown to be endemic in neonatal intensive care units around the world. In this work we studied the genomes of a panel of *S. capitis* isolated from the UK and Germany, we found NRCS-A was common and the most common strain present in neonates. We also identified a group of closely related strains not associated with disease which allowed us to identify genes associated with the higher disease potential of NRCS-A. These included genes involved in phage defence, antimicrobial peptide resistance and metal acquisition and detoxification. This suggests these genes allow NRCS-A to survive in the gut which may help explain the success of this clone and strategies to minimise gut colonisation may be useful in reducing infections caused by NRCS-A.

## Introduction

Non-*aureus* Staphylococci are common commensal bacteria, but many have also been implicated in nosocomial infections including being causative agents of late onset sepsis (LOS) in neonates. LOS is defined as sepsis occurring ≥72 h after birth which can be caused by various bacterial species, the most common being the Non-*aureus* Staphylococci (NAS) [1–4]. LOS increases the length of hospital stay, number of invasive procedures, and provokes more and longer antibiotic treatments, all of which can have negative impacts on the long-term outcomes of new-born babies [1–3, 5].

*Staphylococcus capitis* is one of the most common NAS which are commensal of humans and is regularly cultured from skin or nasal swabs [6]. A particular clone of *S. capitis* ‘NRCS-A’ has been associated with LOS, originally identified in France (the archetypal strain is known as CR01) it has subsequently been found in neonatal intensive care units (NICUs) in 17 countries [4, 7–10]. Infection with NRCS-A has been associated with a higher morbidity and mortality than other NAS [11, 12]. The transmission routes of NRCS-A are not well understood but it has been documented to colonise incubators and equipment and has reduced susceptibility to antiseptics which has been proposed to aid its environmental survival. Unlike other NAS, infection with NRCS-A is not associated with maternal-fetal transmission, and babies born by caesarean section have been shown to be colonised more often than those delivered vaginally [11].

NRCS-A isolates typically have a multidrug resistance profile including methicillin, aminoglycosides and fosfomycin, hetero-resistance to vancomycin (a common treatment for LOS) and decreased susceptibility to the antiseptic chlorhexidine [4, 5, 8, 9]. Many NRCS-A isolates have been found to contain a S*CCmec* mobile genetic element (*SCCmec-SCCcad-SCCars-SCCcop*) which harbours *mecA*, responsible for the methicillin resistance [13–15]. Analysis of genomes of a panel of NRCS-A isolates from various sites and sources suggested the clone emerged in the 1960s before expanding in the 1980s with vancomycin use proposed as a major driver in the selection and evolution of NRCS-A [9]. Decreased susceptibility to vancomycin in this clone has been associated with SNPs in *sarA* and *glnQ* [9].

*S. capitis* infection has only been studied sporadically outside of the neonatal setting but *S. capitis* has been identified in bone and joint infections and, whilst rarely associated with adult disease, NRCS-A has been identified in endocarditis, osteomyelitis and prosthetic joint infections (PJI) in adults [16].

Here we report results analysing the population structure of *S. capitis* isolated as part of a longitudinal survey of NAS from skin and gut swabs taken from babies on NICUs from the UK and Germany [17]. We compared carriage isolates with those from neonatal blood cultures as well as additional reference isolates of NRCS-A and those from healthy adult skin swabs and PJI.

We found that NRCS-A strains were commonly carried by uninfected neonates representing a reservoir of potential infection and identified probable transmission between babies on NICUs. We also identified a group of strains closely related to NRCS-A but without an association with disease. Comparison of the NRCS-A isolates with this group (‘proto NRCS-A’) allowed us to identify the presence of genes encoding phage defence, antimicrobial peptide resistance and metal acquisition/detoxification as key features which we propose may contribute to the success and prevalence of NRCS-A.

## Methods

### Collection of isolates of *S. capitis*

Infants admitted to the NICUs of the Norfolk and Norwich University Hospital (Norwich, UK) or University Children’s Hospital (Lubeck, Germany) over 10 week periods in 2017 or 2018 were enrolled in this study as recently described [17]. Swabs are taken routinely from babies upon admission and throughout their stays in both sites for MRSA surveillance; duplicate swabs were taken for this study and staphylococci isolated. In addition, isolates from positive blood, cerebrospinal fluid, urine, and wound cultures from the study period as well as some collected later.

The UK unit enrolment was between November 2017 and January 2018, and the German enrolment was from January to March 2018. Amies Charcoal Swabs (Thermo Fisher Scientific) were used to isolate bacteria from all infants on admission and weekly until discharge. Swabs from the ear, nose, axilla, groin and gut were taken and streaked on 5 % horse blood agar (Thermo Fisher), prior to incubation at 37 °C for 24 h and final identification of coagulase-negative Staphylococci after sub-culture on mannitol-salt agar (Oxoid, Thermo Fisher Scientific), coagulase testing (MERCK; 75832), and/or MALDI-TOF mass spectrometry (Bruker) as previously described [17]. Isolates were stored on preservation beads at −80 °C (Protect, Technical Service Consultants Ltd) and in 96 deep-well plates in 20 % glycerol at −40 °C. Clinically relevant isolates of *S. capitis* from both the Norwich and Lubeck units were identified by local microbiology departments during the study periods as part of usual practice were also included as well as further anonymised clinical isolates taken from routine blood tests taken from neonates with suspected sepsis at the NNUH NICU collected in 2018 (*n*=7) and between June and May 2022 (*n*=5). In addition to isolates from neonates, a further panel of 15 *S *. *capitis* were taken from a pre-existing Staphylococci collection isolated from adult healthy skin swabs, using Amines charcoal swabs, and clinical isolates taken from adult blood cultures (where infection was suspected) and recovered from PJI [18].

### DNA extraction

Isolates were grown in 1 ml Brain Heart Infusion (BHI, MERCK) broth overnight at 37 °C. Cultures were pelleted and resuspended in 100 µl 0.5 mg ml^−1^ lysostaphin (from *Staphylococcus staphylolyticus,* MERCK) and incubated at 37 °C for a minimum of 1 h. For Illumina sequencing DNA was extracted from the lysate with the Quick-DNA Fungal/Bacterial 96 kit (Cambridge Bioscience), in accordance with the manufacturer’s guidelines. DNA was quantified using the Quant-iT dsDNA HS assay (ThermoFisher), and fluorescence was measured on a FLUOstar Optima plate reader at 480/530 nm (excitation/emission).

For high molecular weight (HMW) DNA, the Quick-DNA HMW MagBead Kit (Zymo) was used with a modified protocol. Starting culture volume was increased to 500 µl and 50 µl of 0.5 mg ml^−1^ lysostaphin (from *Staphylococcus staphylolyticus,* MERCK) was substituted for lysozyme. Isolates were incubated at 37 °C for 1 h prior to adding Proteinase K. DNA was quantified with dsDNA HS Qubit Assay (ThermoFisher).

### Whole genome sequencing

For Illumina whole genome sequencing genomic DNA was normalised to a total of 10 ng and mixed with the tagmentation mix (Illumina) and heated to 55 °C for 15 min. A PCR Kap2G Robust PCR kit (MERCK) and P7 and P5 of Nextera XT Index Kit v2 index primers (Illumina), were used for library preparation, tagmentation mix was added and PCR performed (72 °C for 3 min, 95 °C for 1 min, 14 cycles of 95 °C for 10 s, 55 °C for 20 s and 72 °C for 3 min). Libraries were quantified using the Promega QuantiFluor dsDNA System and run on a GloMax Discover Microplate Reader before being pooled and double-SPRI size selected between 0.5 and 0.7X bead volumes using KAPA Pure Beads (Roche). The final pool was quantified on a Qubit 3.0 instrument and run on a D5000 ScreenTape (Agilent) using the Agilent Tapestation 4200 to calculate the final library pool molarity.

The pool was run at a final concentration of 1.5 pM on an Illumina Nextseq500 instrument using a Mid Output Flowcell (NSQ 500 Mid Output KT v2 [300 CYS] Illumina).

For long read sequencing up to 400 ng of HMW DNA was incubated with Ultra II End-prep reaction buffer and Ultra II End-prep enzyme mix for end repair. The samples then had native barcodes (NB01-24) added with the addition of the NEB Blunt/TA Ligase Master Mix. The libraries were pooled and cleaned using AMPure XP beads, DNA was quantified using dsDNA HS Qubit (ThermoFisher) and Tapestation (Agilent). For the adaptor ligation step, Adapter Mix II (AMX II), NEBNext Quick Ligation Reaction Buffer (5X) and Quick T4 DNA Ligase was added to the pooled library and cleaned using AMPure XP beads. The library was eluted and loaded on to a primed MinION flow cell (R9 4.1) (Oxford Nanopore Technologies). Base calling used Guppy (v6.06) (Oxford Nanopore Technologies).

### Genomic and phylogenetic analysis

Sequence data was processed via a series of pipelines hosted on a Galaxy instance at the Quadram Institute Bioscience. FASTQ files were used to assign a microbial classification and check for contamination for each sample using Centrifuge (v0.15) [19]. Confirmed pure *S. capitis* isolates were then used for further analysis, and assembled with SPAdes (v3.12.0) [20], with default parameters applied, with resulting assemblies analysed for quality with QUAST (v5.0.2) [21] before being annotated with Prokka (v1.14.5) [22]. Isolates where less than 25× genomic coverage was obtained were omitted from the final collection, with an average coverage >50× for the collection.

Long read data was assembled using Flye (v2.5) [23, 24] the Illumina short reads were then mapped to the Flye scaffolds using minimap2 (v2.17) [25, 26] and the sequence files were then polished with two rounds of pilon (v1.20.1) [27].

To identify features associated with NRCS-A isolates, the phylogeny of the *S. capitis* isolates was determined using a core gene alignment created with Roary [28] along with nine available *S. capitis* reference genomes (Table S1). GFF3 files were submitted to Roary (v3.13.0) to determine the core and accessory genomes (this was run with varying cut-offs requiring between 85 and 99 % of isolates to carry a gene to be considered to be in the core before 85 % was used for the final trees after visualisation of results) [28]. A consensus phylogenetic tree was inferred using IQTREE (v 1.6.12) with branch support (1000 bootstrap replicates) [29] from the core gene alignment output from Roary, and then visualised and annotated in iTOL [30]. Associations of gene presence and absence with specific phenotypes of interest were further analysed using Scoary [31].

The presence of antimicrobial resistance and virulence genes were identified using ABRicate (v2.13.2, with 85 % minimum identity, and 90 % minimum coverage filters applied) [32] and ARIBA [33] using the CARD database (v3.1.1) [34].

To identify SNPs, FASTQ files were processed by Snippy (v4.4.3), and Snippy-core (v4.4.3) used to generate a core alignment FASTA which was then analysed by snp-dist (v0.6.3) [35, 36]. The reference genome used for all Snippy tools was the NRCS-A CR05 genome (NZ_CTEO01000001.1). The Snippy-core alignment was then used to create a phylogeny based on core SNPs and run through IQTREE [29], the resulting maximum likelihood tree was visualised using iTOL [30] (Fig. S1, available in the online version of this article).

Homologues of specific sequences of interest were identified using BLASTn, sequence data for the *SCCmec-SCCcad-SCCars-SCCcop* (KF049201.1) mobile element was used as a reference to identify other similar sequences in the collection using megablast (v2.10.1; default settings) [37, 38]. The NCBI protein database was searched for homologues to *nsr* (CDI72769.1) and *tarJ* (CDI72761.1) with BLASTp [38]. Alignment of CRISPR-Cas Type-III-A genes from different strains was assessed by taking a reference sequence from *S. capitis* CR05 and creating a Snippy-core full alignment which was then submitted to ClustalW (v1.91) [39]. Long read sequencing data for *S. capitis* genomes were directly compared using BLASTn pairwise alignment [40] and visualised using Artemis Comparison Tool [41].

### Antimicrobial susceptibility testing

The susceptibility of all *S. capitis* isolates to the biocides chlorhexidine gluconate (CHX) and octenidine hydrochloride (OCT) and antibiotics, benzylpenicillin (PEN), cefotaxime (CEF), daptomycin (DAP), gentamicin (GEN), fusidic acid (FUS) and vancomycin (VAN) were determined in Mueller-Hinton agar (for daptomycin, Ca^2+^ was added to a final of concentration 50 mg l^−1^) (Sigma-Aldrich) in accordance with EUCAST guidelines for agar dilution. Plates were inoculated with ~10^4^ cells diluted from overnight cultures grown in Mueller-Hinton broth (Sigma-Aldrich) using a multi-point inoculator (Denley). *Staphylococcus aureus* controls were run on all the plates, ST 239 (‘TW20’) and NCTC 8532 (‘F77’) were used on the biocide plates and ATCC 29213 for the antibiotic plates. The plates were incubated at 37 °C for 24 h before minimum inhibitory concentrations (MICs) were determined. MIC_50_ and MIC_90_ were determined by identifying the MIC which inhibits 50 % or 90 % of the collection. Clinical breakpoints from EUCAST [42] were used where applicable and cut off points for CHX and OCT were set at 4 µg ml^−1^ and 2 µg ml^−1^ respectively, as defined by H. L. Htun *et al*. [43]. Statistical analysis was performed using Graph Pad Prism (v5.04). For analysis of more than two groups a one-way ANOVA Kruskal-Wallis, non-parametric test was used.

### Sensitivity to pH

Two randomly selected isolates from each of the non-NRCS-A, proto NRCS-A and NRCS-A groups were used to test for ability to grow at different pH values (pH range 3.5–10). Overnight cultures of each were used to inoculate Biolog plates in duplicate (PM10 MicroPlate pH, Biolog, UK) in accordance with the manufacturer’s protocol and respiratory activity recorded based on reduction of a redox dye mix produced by the manufacturer. Values are recorded as ‘Omnilog Units’ (OU), by measuring changes in light transmission [44, 45], in each sample over 24 h automatically using the OmniLog reader (Biolog, UK).

## Results

### *Staphylococcus capitis* were isolated from various body sites of babies on NICUs

Routine swabs were taken from a total of 159 babies across their stay in NICU, *S. capitis* was isolated from 55 babies (34.5 %) admitted to NICUs in the UK and Germany yielding a total of 102 *S *. *capitis* isolates (UK *n*=90, German *n*=14) from four different body sites (ear *n*=47; gut *n*=13; nose *n*=17; skin *n*=27). A further 12 isolates were from neonates with infection, 11 from blood and one from skin. A total of 15 additional *S. capitis* were collected from adults including from prosthetic joint infection (*n*=3), blood (*n*=5) healthy skin swabs (*n*=6) and one from an abdominal swab. All 129 strains were whole genome sequenced giving an average genome size of 2.52 Mbp with an average GC content of 32.86 %. A further nine previously published reference genomes were also included in later analysis of genomic relationships.

### Antiseptic susceptibility differed between isolates from different geographical sites

**Fig. 1.**
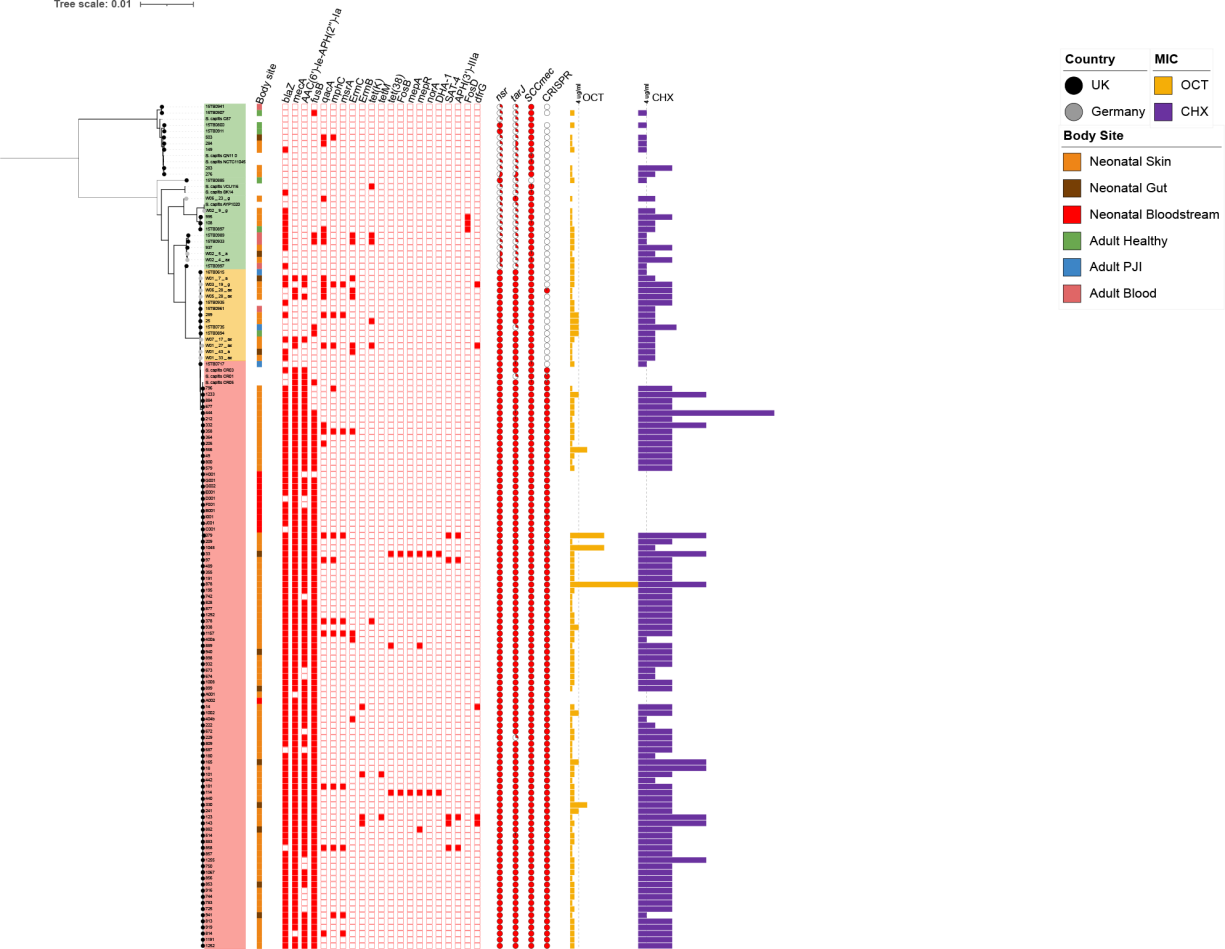
Population structure of *S. capitis *. A consensus tree based on core gene alignment from Roary (bootstrap replicates 1000). Three groups are indicated; non NRCS-A isolates are highlighted in green, proto NRCS-A in orange, and NRCS-A, in pink. Country of isolation is indicated by the colour of tips; Germany (grey circles), UK (black circles). Body site of isolation are shown by coloured boxes; orange – neonatal skin, brown – neontal gut, red – neonatal bloodstream, green – healthy adult skin, blue – adult prosthetic joint, pink – adult bloodsteam. Antimicrobial resistance (AMR) genes are represented by red boxes (blank boxes represent absence of the corresponding gene). The percentage identity of *nsr* and *tarJ* in each strain (compared to the reference sequences present in CR01 and CR05 respectively using BLASTp) are shown as pie charts. MIC data for chlorhexidine (CHX, purple bars) and octenidine (OCT, yellow bars) are shown with the dotted lines representing 4 µg ml^−1^ of OCT or CHX. Branches where bootstrap support was >80 % are shown in bold.

All the *S. capitis* collected were tested for antiseptic and antibiotic susceptibility (Table S2) [17]. The *S. capitis* isolates demonstrated greater sensitivity to octenidine than chlorhexidine (MIC^50^ of 2 µg ml^−1^ and 16 µg ml^−1^, and MIC^90^ being 4 µg ml^−1^ and 32 µg ml^−1^ respectively) (Fig. 1). There was no significant (*P*>0.05) difference found in susceptibility of isolates from different body sites, but the MIC^50^ values for both octenidine and chlorhexidine in the German isolates (OCT 1 µg ml^−1^; CHX 8 µg ml^−1^) were lower than those of the UK isolates (OCT 2 µg ml^−1^; CHX 16 µg ml^−1^). As well as the antiseptics, UK isolates had higher MIC^50^ values for gentamicin, penicillin and fusidic acid. The largest discrepancy being for fusidic acid where the MIC^50^ increased from 0.625 µg ml^−1^ for the German population to 4 µg ml^−1^ in UK isolates. Seven adult isolates showed resistance to daptomycin (defined as an MIC ≥1 µg ml^−1^, [15]) but this was not seen in any neonatal isolates. No vancomycin resistance was observed, however nearly a quarter of the isolates exhibited intermediate susceptibility to vancomycin (*n*=29) indicated by a MIC of 2 µg ml^−1^ [46], with the majority of these (23/29) being from NICU.

### Phylogenetic characterisation of *S. capitis* identified NRCS-A and related isolates in a clade distinct to other strains

Phylogenetic trees were initially based on core SNP alignment of all strains compared against reference genome CR05 (Fig. S1), then on the core gene alignment output by Roary of all isolates in the panel (including nine *S. capitis* reference genomes [Table S1, Fig. 1]). Both trees identified a similar population structure with a clade containing NRCS-A strains identified based on similarity to known NRCS-A reference strains (CR01, 03 and 05) and by the presence of the two genes *nsr* and *tarJ,* commonly used as markers for this clone [47]. The trees showed a diverse group of non-NRCS-A clones and then a clonal group which all carried *nsr* and *tarJ*, this included the NRCS-A strains but could also be divided into two further groups based on carriage of a CRISPR system.

**The ‘non-NRCS-A’ -** This group (Fig. 1, green, *n*=27) contained no known NRCS-A strains but did include other *S. capitis* NCBI reference genomes and isolates from adults as well as some NICU isolates. Isolates in this group were and generally showed a lower tolerance to OCT and CHX (OCT MIC_50_=2 µg ml^−1^, CHX MIC_50_=8 µg ml^−1^; Table 1) than NRCS-A strains. They were also susceptible to fusidic acid with *fusB* being absent in all but one strain (Table S3). Of these non-NRCS-A isolates 12 were collected from the NICU (four German, eight UK), nine from healthy adult skin swabs (*n*=5) or blood cultures (*n*=4). This group also included six non-NRCS-A reference strains (* S. capitis* strains AYP1020, C87, QN1, SK14, VCU116 and NCTC11045 [Table S1]).

**The 'NRCS-A' -** This group (Fig. 1, red, *n*=95) was the largest and contained most isolates as well as NRCS-A reference strains, *S. capitis* CR01, CR03 and CR05 along with the remaining *S. capitis* isolated from the NICU (Fig. 1). No isolates from adults were found in this group. Tolerance to OCT and CHX was marginally higher than isolates from proto NRCS-A (OCT MIC_50_=2 µg ml^−1^, CHX MIC_50_=16 µg ml^−1^: Table 1). NRCS-A was defined by isolates carrying multiple AMR genes including *blaZ* (β-lactamase)*, fusB* (fusidic acid resistance)*, AAC(6’)-la-APH(2’)-la* (aminoglycoside resistance) and *mecA* (penicillin/methicillin resistance) and contained the *nsr* and *tarJ* genes as well as a CRISPR-Cas Type III-A system (Fig. 1 and Table S3).

**The 'Proto NRCS-A' -** This group (Fig. 1, orange, *n*=16) strains contained *nsr* and *tarJ* but no CRISPR system. These strains carried fewer AMR genes than the NRCS-A isolates and typically lacked *fusB*. These strains are closely related to the NRCS-A group and were considered most likely to be ancestral to the NRCS-A which then gained CRISPR and resistance cassettes, however this remains a prediction. This proto-NRCS-A group had OCT and CHX susceptibilities lower than NRCS-A (OCT MIC_50_=2 µg ml^−1^, CHX MIC_50_=8 µg ml^−1^: Table 1). Along with ten isolates from NICU, the remaining isolates were sourced from adult PJI (*n*=3), blood (*n*=1), an abdominal swab and a healthy adult skin swab.

**Table 1.** MIC^50^- and MIC^90^ values of antimicrobials against the *S. capitis* groups

	OCT		CHX		VAN		GEN		PEN		CEF		CIP		DAP		FUS	
	MIC^50^	MIC^90^	MIC^50^	MIC^90^	MIC^50^	MIC^90^	MIC^50^	MIC^90^	MIC^50^	MIC^90^	MIC^50^	MIC^90^	MIC^50^	MIC^90^	MIC^50^	MIC^90^	MIC^50^	MIC^90^
Non NRCS-A	2	2	8	16	1	2	0.064	32	0.5	8	1	4	0.5	4	0.5	2	2	2
Proto NRCS-A	2	4	8	16	1	2	1	32	2	32	1	4	0.25	0.5	0.5	1	0.064	2
NRCS-A	2	4	16	32	1	2	16	32	8	126	4	4	0.5	4	0.25	1	4	4
Overall	2	4	16	16	1	2	8	32	4	126	4	4	0.5	4	0.5	1	2	4
UK	2	4	16	32	1	2	8	32	4	126	4	4	0.5	4	0.5	1	4	4
German	1	2	8	16	1	1	8	32	4	126	4	4	0.5	4	0.5	1	2	2

CEF, Cefotaxime; CHX, Chlorhexidine gluconate; CIP, Ciprofloxacin; DAP, Daptomycin; FUS, Fusidic Acid; GEN, Gentamicin; OCT, Octenidine; PEN, Benzylpenicillin; VAN, Vancomycin.

**Fig. 2.**
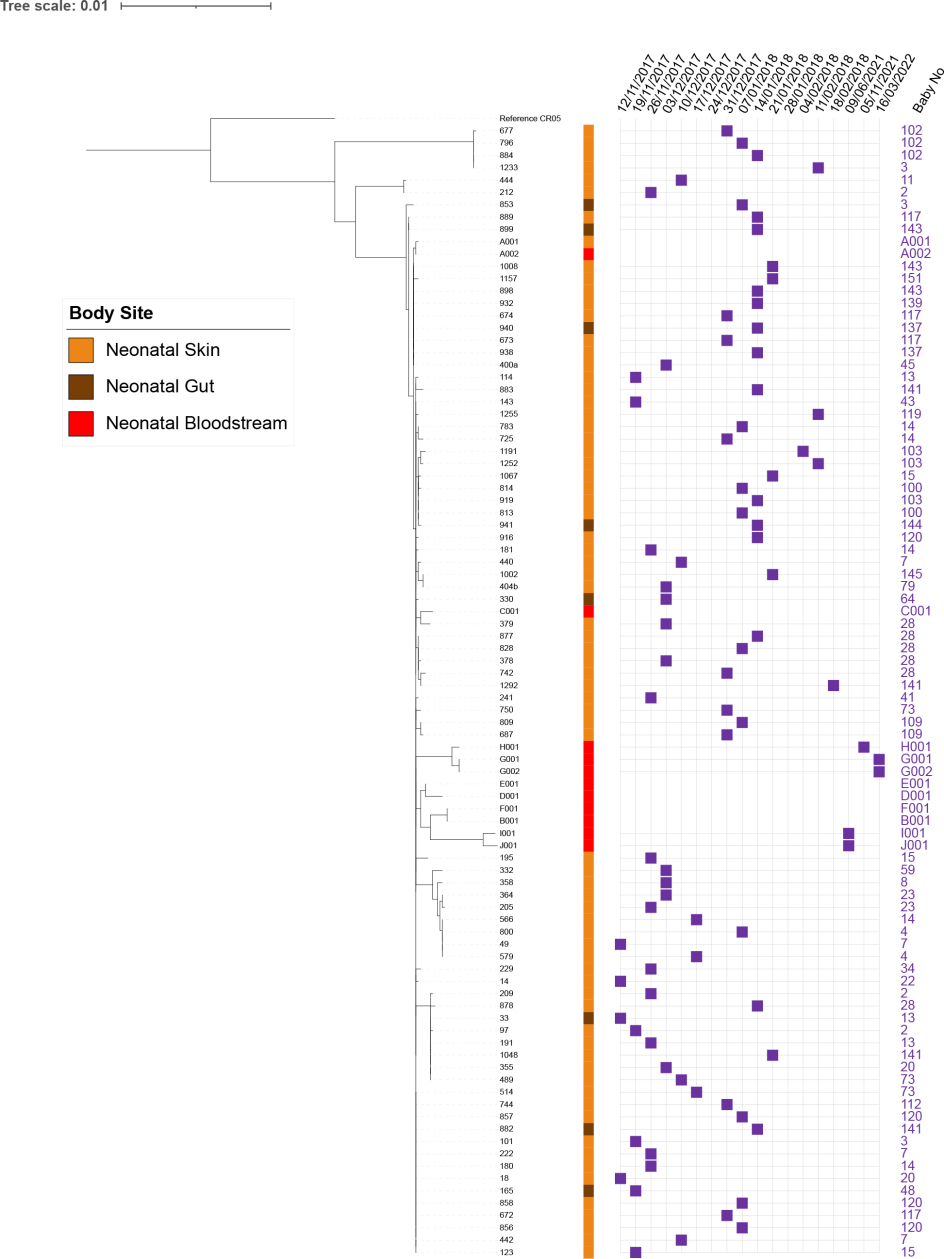
Detailed phylogeny of NRCS-A based on core SNP alignment. The week of isolation is indicated by purple squares for all UK isolates and sources of each isolate are indicated by coloured squares (neonatal skin=orange; neonatal gut=brown, neonatal bloodstream=red).

Multiple isolates of *S. capitis* were isolated on different sampling dates from 24 separate babies (Fig. 2) with the remaining 40 babies only yielding a single isolate. Only four of 64 babies carried non-NRCS-A *S. capitis* strains alone (babies 48, 58, 94 and 146, Fig. 2). The remaining 60 babies carried NRCS-A isolates (Table S2). Three babies (baby 8, 59, and 137) yielded *S. capitis* isolates from two of the three groups (non NRCS-A one and NRCS-A), and one baby had two distinct isolates from the proto NRCS-A group (baby 28), and another yielded one non-NRCS-A and one proto NRCS-A isolate (Table S2).

The number of SNPs distinguishing all 138 isolates was determined using snippy with UK NRCS-A CR05 as a reference. The mean (standard deviation) number of SNPs seen between isolates in the NRCS-A, and proto NRCS-A groups were 115.47 (0.6) and 265 (7.3) respectively (Fig. S2 and Table S4). The non-NRCS-A isolates contained an average of 23 084.5 (3263.2) SNPs, a much higher number confirming that these strains are genetically distant from the NRCS-A isolates and more diverse.

When comparing only the 92 NRCS-A genomes there was a maximum of 37 core SNPs between each other (mean aligned core genome: 2401507 bp), these isolates were all from the UK NICU (Table S4). This high degree of relatedness of isolates in one UK NICU suggests a recent common ancestor with transition across the unit.

### Evidence for transmission within the NICU

Closely related isolates persisting over time were observed within the UK NICU (Fig. 2), with near identical isolates being found on different babies and over time. On analysing relationships between isolates taken longitudinally from babies (Fig. 2), this revealed a cluster of eight isolates (209, 878, 33, 97, 191, 1048, 355, 489) differentiated by only three SNPS, (Table S5) recovered over 70 days, from six different babies (Fig. 2). This suggests that there is transmission of NRCS-A within the NICU environment and indicates persistence of the NRCS-A clone over time. Closely related isolates from bloodstream infection from babies on the same unit were isolated years after the initial screen demonstrating this clone is present within the unit and subject to little genomic change (Fig. 2).

### Differences in gene content distinguishing groups

**Fig. 3.**
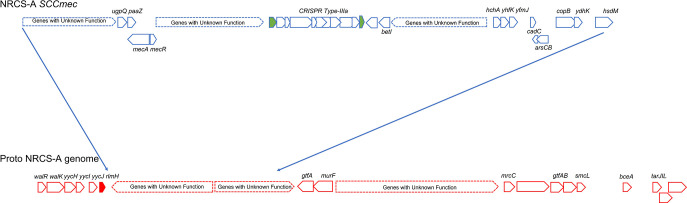
Visualisation of the SCCmec mobile genetic element including the CRISPR genes found in the NRCS-A group (isolate 440), and site of insertion into the Proto-NRCS-A genome down stream of rlmH (previously known as orfX, filled red). CRISPR repeat regions are filled green, sequence data attained from longread MinION polished with Illumina reads.

The collection was analysed by Roary [28], to identify genes that were either present or absent and differentiated the groups, ‘Scoary’ was then used to calculate statistical associations between the presence and absence of genes, clade membership and a series of phenotypes [31]. Notably the genes being most significantly associated with the NRCS-A group were the CRISPR genes, all NRCS-A isolates harboured a complete CRISPR-Cas Type-III-A system. None of the closely related proto NRCS-A isolates harboured any CRISPR Cas Type-III-A genes. These Cas Type-III-A genes are inserted in the TypeV *SCCmec-SCCcad/ars/cop* element inserted at the typical site adjacent to *rlmH* (*orfX*) (Fig. 3). Two genes *paaZ* and *ugpQ,* along with *dut* are closely linked to *mecA,* and were therefore also strongly associated with the NRCS-A group.

Scoary analysis also identified groups of other genes significantly associated (corrected *P*= <0.005) with the NRCS-A isolates. Many of these genes were annotated as being of unknown function and clustered together in the genomes. Further analysis of these genes, using Blastn identified several as being putative phage terminases which are inserted in-between *yfnK* and *murI,* immediately downstream from *frdA, uvrC* and *mutS*. Scoary also identified *mcrC* in NRCS-A isolates which is involved in regulation of degrading methylated bacteriophage DNA [48] (Table S6).

Previous research by P. Martins Simões *et al*. [47] suggested the presence of *nsr* and *tarJ* as a defining characteristic of the NRCS-A clone, and both these genes were present in both NRCS-A and proto NRCS-A isolates. The *tarLIJ* locus encodes a ribulose-5-phosphate reductase which are involved in teichoic acid biosynthesis [16, 47]. These were significantly associated with the NRCS-A group, parts of the operon were found in non-NRCS-A isolates but were usually incomplete (Fig. 1, Table S6). All NRCS-A and proto NRCS-A isolates had *tarJ* compared to only 3.7 % of non-NRCS-A isolates (Fig. 1). The *nsr* gene associated with nisin resistance was identified in both NRCS-A and proto NRCS-A isolates, and *kdpDC* was also significantly over-represented in these groups which encodes a potassium pump often encoded with *nsr* [47].

**Fig. 4.**
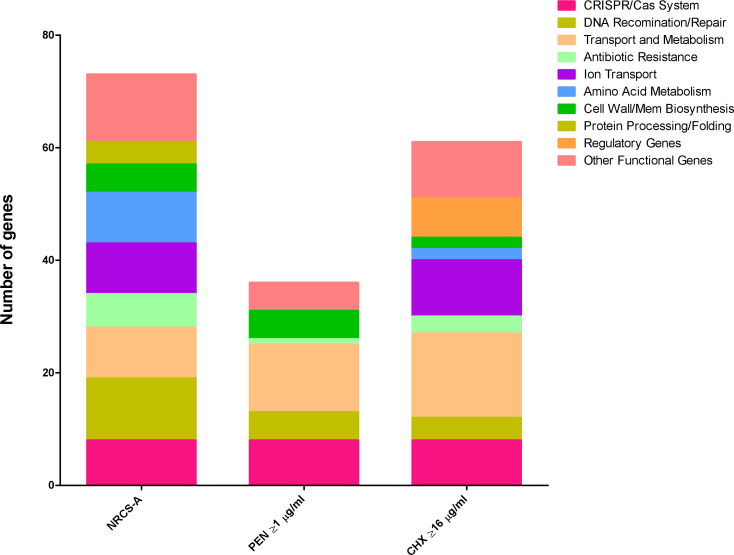
Functional groups with significantly altered numbers of genes present that differentiate either association with the NRCS-A isolates compared to other groups, isolates with penicillin MICs>1 µg ml^−1^ or CHX MICs>16 µg ml^−1^. Significance was calculated by Scoary using adjusted *P* values determined using Benjamini-Hochberg’s method [31]. Raw data is given in Table S6.

There was a significant increase in the presence of genes involved in metal transport in the NRCS-A group compared to the others (Fig. 4, Table S6). ABC permeases *cntABCDEFKL, nikC* and *oppDF* were all identified in NRCS-A strains and are involved in the acquisition of Zn and Ni [49]. Further genes involved in metal detoxification were also seen in NRCS-A, the *copB, copAZ, arsBC* and *cadC* systems were over-represented in this group. These have all been previously identified as part of an SCC*mec* element (*SCCmec-SCCcad/ars/cop*) also containing the CRISPR-Cas Type III-A system in NCRS-A isolates. As well as genes associated with this element, the NRCS-A isolates could also be distinguished from the proto-NRCS-A strains by the presence of *mntH* (Table S6).

To compare conservation of the *SCCmec* element seen in all the isolates, the S*CCmec-SCC-cad/ars/cop* sequence of CR01 (GenBank KF049201.1) was compared to all the *S. capitis* isolates using snippy and snp-dist [35, 36]. All isolates and references except for VCUII6 aligned to the S*CCmec-SCC-cad/ars/cop* reference. The NRCS-A isolates, on average, had 12.3±0.5 SNPs, which increased to 64.4±11.1 in non-NRCS-A and 190.9±22.5 in proto NRCS-A isolates. The higher divergence in the proto NRCS-A group in the *SCCmec* elements is surprising given its closer relationship to the NRCS-A group according to the core genome phylogeny.

Further analysis of the CRISPR-Cas Type III-A system showed that the genes were highly conserved when aligned. In all isolates it was possible to identify direct repeat (DR) sequences (beginning with GATAACT), and there were three different DRs present in the isolates as previously observed in CR01 [50]. In a full SNP ClustalW alignment DR5 (four times) and DR6 (three times) were found before the Cas genes, and then two repeats of DR8 towards the end of the alignment after the CRISPR genes (Fig. 3) [50].

**Fig. 5.**
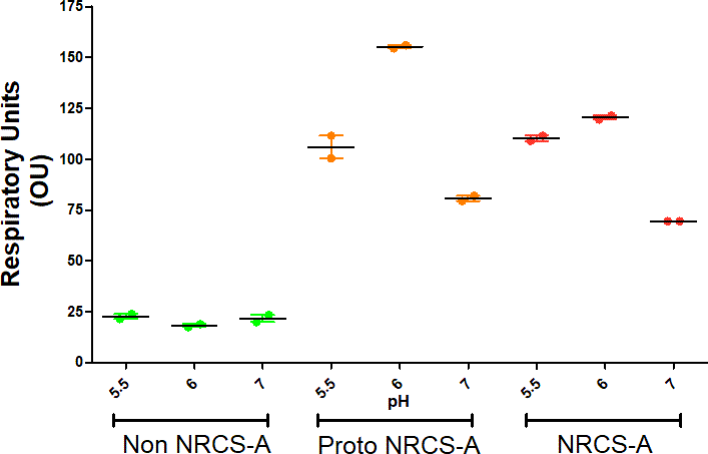
Growth of *S. capitis* in different pH (pH 7, 6 and 5.5), showing growth at final time point of 48 h from PM10 MicroPlate. Points indicate mean values (*n*=4) of respiratory activity (measured in Omnilog Units) and bars the standard error.

Previously it has been suggested that NRCS-A isolates are adapted to survive in both the gut and on the skin, two environments where pH differs. The natural skin surface pH is variable and been shown to fluctuate between pH 5-6 [51], whereas the pH increases from pH 6 in the duodenum to 7.4 in the ilieum and falls to pH 5.7 in the caecum [52]. We observed that growth of both proto NRCS-A and NRCS-A isolates at pH 5.5 was higher compared to non-NRCS-A isolates (Fig. 5). Growth at pH 6 and pH 5.5 was also marginally higher compared to pH 7 in both the NRCS-A and proto NRCS-A isolates (Fig. 5). No growth was detected between pH 8–10, or pH 3–3.5 in any of the isolates tested giving a small range of pH that are favourable to the proto and NRCS-A strains compared with others.

### Carriage of AMR genes varied between non NRCS-A and NRCS-A

**Fig. 6.**
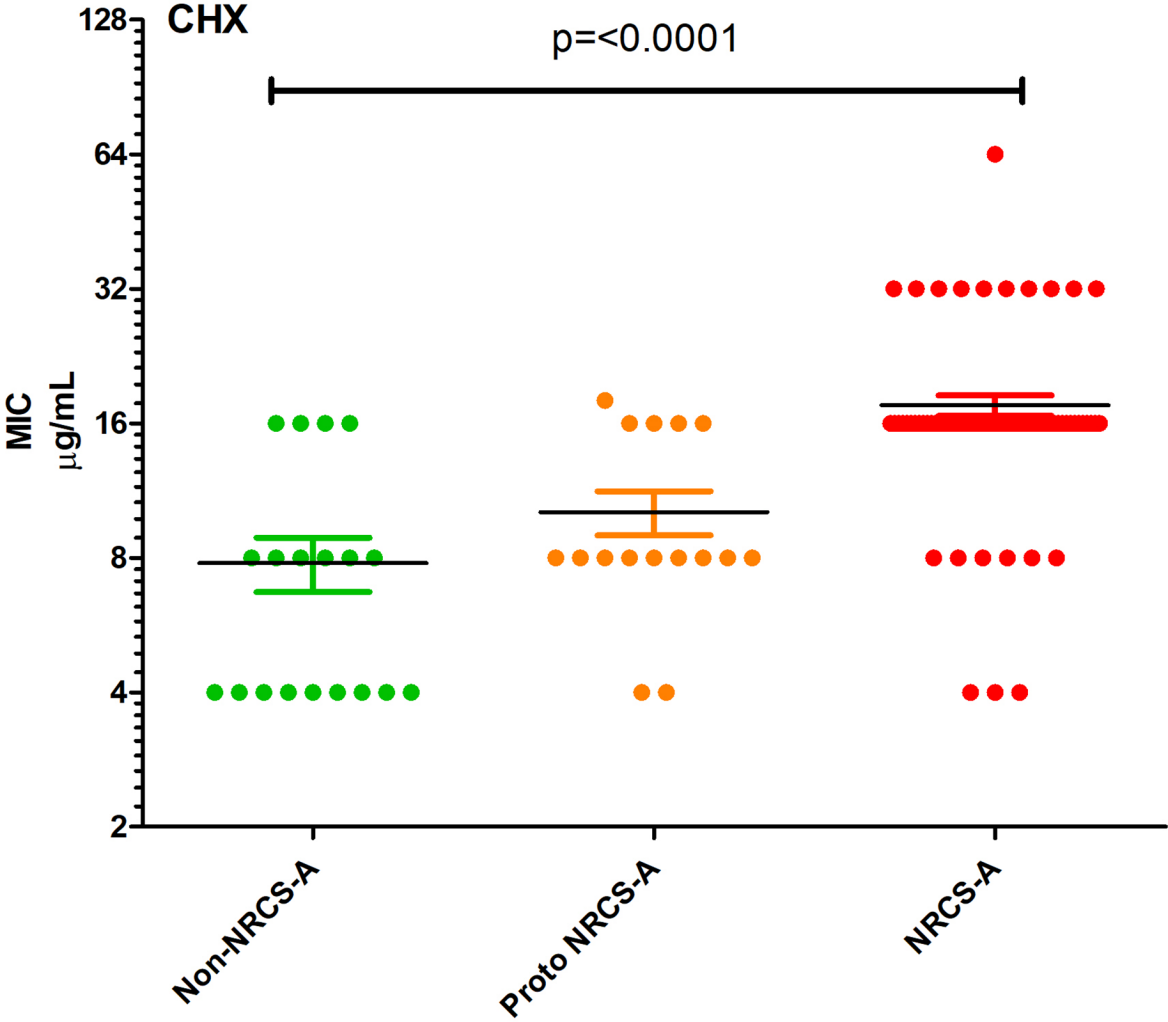
Chlorhexidine susceptibility of the three groups of *S. capitis *. Points represent values from individual strains, horizontal lines indicate the means with bars showing the standard error of the mean.

All isolates contained *lmrS, norA, arlR* and *dfrC*, and all but one isolate contained *mgrA*. NRCS-A was defined by the additional presence of *blaZ, mecA, ACC(6’)-le(APH)(2’)-la* and *fusB* in the majority of isolates whereas proto NRCS-A had a smaller number of AMR genes which were interspersed across these strains without a consistent pattern (Fig. 1, Table S3). Analysis of the pangenomes with Scoary was used to identify genes correlating with susceptibility to different antimicrobials. Unsurprisingly *mecA* was strongly associated with high cefotaxime MICs (4 µg ml^−1^, Table S6) and carriage of *blaZ* was also associated with penicillin resistance (≥1 µg ml^−1^, *P*=0.005), however CRISPR genes (*P*=0.002) and other genes commonly found on the S*CCmec-SCC-cad/ars/cop* MGE were more significant (Fig. 4, Table S6). The *qac* family of genes has previously been implicated in tolerance to antiseptics, *qacA* was detected but only present in 22/138 (16 %) isolates and there was no correlation with the presence of *qacA* and CHX tolerance. Carriage of *qacA* was only observed in 16.25 % of isolates with high chlorhexidine MICs≥16 µg ml^−1^ (Fig. 1). For isolates that had an MIC to chlorhexidine of ≥16 µg ml^−1^ the genes mostly associated with this tolerance were the CRISPR-Cas Type III-A (Supplementary Table 6). This is a marker of NRCS-A isolates which had significantly higher chlorhexidine MICs (Fig. 6) although there was no obvious genetic basis for this. Scoary was unable to identify any significant genes associated with octenidine susceptibility.

The classic antibiogram for NRCS-A isolates includes being resistant to fusidic acid [53], with the clinical breakpoint being ≥0.5 µg ml^−1^ for NAS [42]. Based on this criterion 79 % of our collection tested were clinically resistant and carriage of *fusB* was seen in 78.75 % of these resistant isolates, leaving 17 isolates with no known mechanism of fusidic acid resistance. The presence of *fusB* was mainly observed in NRCS-A, where there was a small increase in tolerance to fusidic acid, with the mean MIC being 2.95 (SD: 0.155) µg ml^−1^, higher than non-NRCS-A isolates (1.40, SD: 0.301 µg ml^−1^). The UK isolates had a higher median fusidic acid MIC (4 µg ml^−1^) than the German isolates (1.5 µg ml^−1^) (*P* = <0.01). Penicillin resistance encoded by *mecA* was seen in 86 % of the isolates with an MIC ≥2 µg ml^−1^ (Tables S2 and S3), and carriage of *ACC(6’)-la-APH(2’)-la* was seen in 73 % of isolates with an MIC ≥1 µg ml^−1^ of gentamicin. No genes were associated with ciprofloxacin resistance although the main mechanism of resistance to this agent is target site mutation within *gyrA* rather than the presence of a gene.

*S. capitis* NRCS-A are often referred to as demonstrating hetero-resistance to vancomycin, indicated by a MIC of 2 µg ml^−1^ [46]. Despite 23.5 % of the isolates having an MIC of 2 µg ml^−1^ there was no presence of *vanA* apart from in one isolate (with an MIC of 1 µg ml^−1^) or any other significant genes associated with vancomycin resistance.

## Discussion

Even with the increased focus on their prevention, nosocomial infections remain a major cause of disease and death in hospitals. Transmission of bacterial pathogens can occur via multiple routes and vectors with both health care workers and the environment sources of infection for many pathogens including *Staphylococcus aureus, Pseudomonas aeruginosa* and Enterobacteriaceae [54, 55]. The wide dissemination of the NRCS-A clone within NICUs around the world has been well established although the adaptations allowing this remain unclear. One suggestion is that NRCS-A isolates are adapted to survival in the gut which provides an additional reservoir to the skin and may allow persistent colonisation, even when skin organisms are lost during antisepsis [10]. Carriage of the *nsr* gene responsible for nisin resistance has been proposed to aid survival in the gut where this antimicrobial peptide is produced [47]. Consistent with this hypothesis we documented carriage of NRCS-A on both the skin of neonates and in the gut, and this was much more common than for other groups of *S. capitis* (Fig. 1). We did observe that the *nsr* gene is present in NRCS-A isolates but not in more distantly related strains. Interestingly we identified a group of isolates closely related to NCRS-A, the ‘proto NRCS-A’ strains which did carry *nsr* but not the CRISPR system, and these were isolated from the gut less often (Fig. 1). Whilst it is not possible to define with certainty the evolutionary events separating the proto and NRCS-A strains it seems likely the proto group are ancestral and NRCS-A isolates gained the CRISPR island. Given the differences between the proto and NRCS-A groups seem to be mainly due to gene gain by NRCS-A a Bayesian analysis of potential time of divergence was likely to be inaccurate so we did not feel able to reliably estimate when this may have occurred with any certainty.

We observed that both the proto NRCS-A and NRCS-A isolates, which both possess *nsr* and *tarJ* had increased growth in pH 6–5.5 cultures compared to more divergent strains (Fig. 4), which may help survival in the gastrointestinal tract. No NRCS-A clones were isolated from the German NICU, but both proto-NRCS-A and non-NRCS-A strains were identified. Some of these German proto NRCS-A isolates harboured some characteristics of the NRCS-A group, but they were not consistent, such as the presence of the *blaZ, mecA, fusB* and AAC(6’)-le-APH(2’)-la genes frequently associated with the NRCS-A isolates.

### Metal acquisition and survival

Analysis of the pangenomes of each group identified other genes that may aid the survival of NRCS-A in the NICU. In NRCS-A isolates there was a marked increase in carriage of genes associated with metal chelation (Fig. 4, Table S4). Availability of many metals is limited *in vivo* and in the gut metals are precious commodities. Metal chelators can also influence virulence, for example, the nickel/cobalt transporter *nixA* in *S. aureus* has been implicated in an increase in virulence in UTI and kidney infection and is required for urease activity in mice [56]. Other metal related genes found in NRCS-A were *mntH*, a metal transporter found to minimise impact of Mn and Zn starvation [57]*,* and *cntABCDEF* which acts as an transport system for staphylopine, (encoded by *cntKLM*) a staphylococcal zincophore involved in Zn acquisition which can also bind to Cu, Co and Ni [49, 58]. These high affinity chelators and zincophores have been shown to be able to outcompete human calprotectin (a Zn chelator excreted by neutrophils) to evade nutritional immunity. The presence of *cntABCDEF cntKLM, nixA* and *mntH* may indicate that even in nutritionally deprived environments, the NRCS-A isolates are able to acquire essential metal ions and survive in a gut environment. Similarly, the presence of *copA, cad,* and *ars,* encoded within the *SCCmec-SCCcad/ars/cop* element in NRCS-A provide defence against toxic metals by encoding efflux systems. Together these data suggest that an enhanced ability to scavenge and survive metal exposure may be crucial for the success of NRCS-A and supports the theory that gut colonisation is a key strategy exploited by this clone.

### Phage defence

If NRCS-A has developed a lifestyle which includes enhanced ability to colonise the gut then it will also need to adapt to attack from phage, which are prevalent in this environment. In *Escherichia coli* the *mcrBC* genes provide protection from phage and McrC activates McrB to degrade methylated bacteriophage DNA [48]. MrcC was seen in our collection but *mcrB* was not detected and it is unclear what impact *mcrC* alone may have. Other mechanisms of phage defence observed in the NRCS-A isolates were the CRISPR-associated genes (Cas), used in defence against MGEs, viruses and plasmids [59, 60]. CRISPR-Cas systems show low abundance in most NAS although they have been documented in the genomes of *S. aureus* strains as well as in *S. epidermidis, S. schleiferi, S. haemolyticus* and *S. lugdunensis *. Carriage of a CRISPR-Cas system is a known feature of NRCS-A *S. capitis* and CRISPR-Type III-A Cas genes were identified in all NRCS-A isolates. This was the main defining feature differentiating the proto NRCS-A and the NRCS-A groups, however when this system was acquired by NCRS-A remains unclear. The direct repeats identified are similar to those previously seen in CR01 and CR03 which both contain the conserved CCCC and GGGG motifs which are thought to form hairpin structures [60]. The highly conserved regions suggest that a common ancestor is responsible for the acquisition of the CRISPR-Cas Type-III-A genes.

### Antiseptic tolerance

Another defining feature of the NRCS-A strains was increased tolerance of chlorhexidine (Fig. 6), this was not associated with carriage of *qac* genes, and the mechanism remains unclear. This increased ability to survive chlorhexidine exposure may allow both survival of skin antiseptics but also environmental decontamination where chlorhexidine is a common part of cleaning and disinfection routines for incubators and medical equipment. There was no elevation in tolerance to octenidine, which is the main alternative for antisepsis in neonates [17].

### Stability and persistence of NRCS-A

The NRCS-A isolates compared here were very similar including those separated by geography (UK and German isolates) and time (isolates from the collection period and subsequent clinical isolates years apart in isolation (Fig. S2, available in the online Supplementary Material), with no more than 37 SNPs separating strains in the core genome. We provide evidence for transfer between babies on the NICU (Fig. 2), and previous studies identifying NRCS-A in the NICU environment and on different surfaces or medical implements following clusters of cases of LOS caused by NRCS-A have suggested a role for the environment in transmission. The isolates from carriage and the ten from neonatal blood cultures were highly similar (Fig. 1) supporting the idea that carriage acts as a reservoir for infection although given the low numbers of cases of infection carriage by neonates in a nosocomial setting does not usually result in infection.

## Conclusions

We have identified that carriage of the NRCS-A clone is abundant on the skin and gut of uninfected neonates and that they are likely to be transferred within NICUs. NRCS-A isolates separated in time and space showed little genetic variation and carriage isolates were indistinguishable to those from blood culture, suggesting carriage can be a precursor to infection. We identified a closely related proto-NRCS-A group which was less associated with gut carriage, did not possess the CRISPR-Cas III-A system, demonstrated lower antimicrobial resistance and chlorhexidine tolerance, and carried fewer metal acquisition or detoxification genes. Our data supports the idea that gut colonisation is a key survival strategy of NRCS-A and expands our understanding of the likely mechanisms employed by this clone to survive on skin, in the environment and in the gut. This opens the possibility to develop a probiotic approach to hinder colonisation of NCRS-A based upon nutritional immunity where strains that sequester essential metals may prove beneficial to the infant gut. Recent deployment of a probiotic product into the NNUH NICU has been used to reduce incidence of necrotising enterocolitis with success and was also associated with reduced episodes of infection with CoNS [61]. More work can help develop strategies to identify reservoirs and transmission routes of NRCS-A and to eradicate carriage from neonates to prevent infection which can have serious consequences.

## Supplementary Data

Supplementary material 1

Supplementary material 2

Supplementary material 3
