# Supplementary material for: Characterisation of neonatal Staphylococcus capitis NRCS-A isolates compared with non NRCS-A Staphylococcus capitis from neonates and adults
Source: Microb Genom. 2023 Oct 4;9(10):001106. doi: 10.1099/mgen.0.001106 (PMC10634448; doi:10.1099/mgen.0.001106)
Supplement: Supplementary material 1 [file mgen-9-1106-s001.pdf]

Supplementary figures

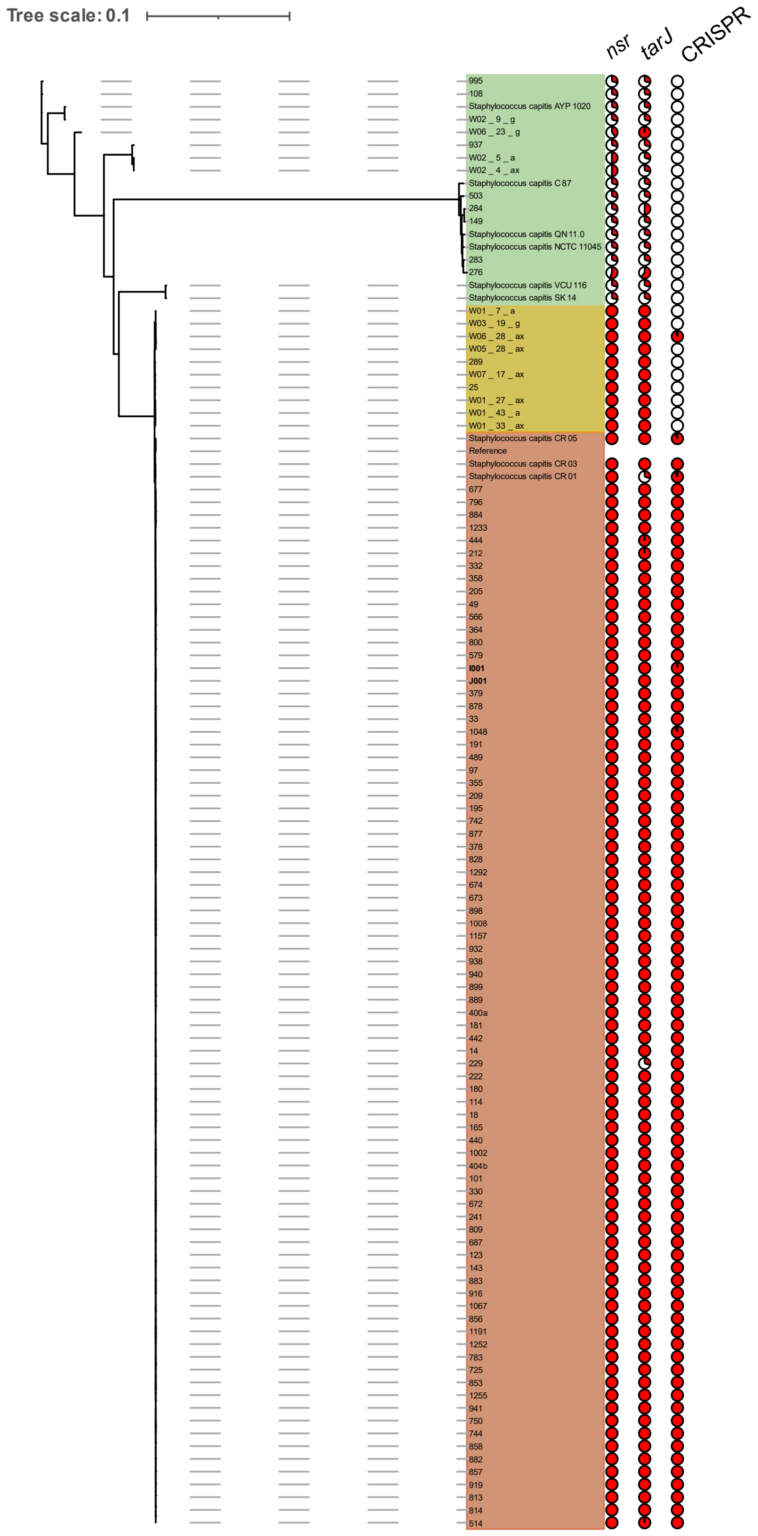

Supplementary figure 1. Maximum likelihood phylogenetic tree of all isolates based on core SNPs compared against NRCS-A CR05 (NZ_CTEO01000001.1).

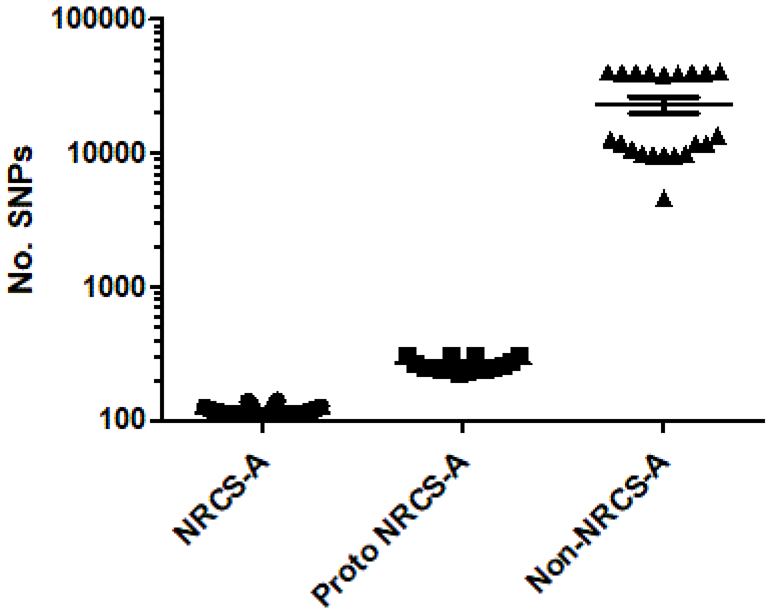

Supplementary figure 2. Numbers of SNPs in strains from each group compared against NRCS-A CR05 (NZ_CTEO01000001.1)
